# Serum antioxidant capacity and peroxide level of seven healthy subjects after consumption of different foods

**DOI:** 10.1016/j.dib.2016.10.028

**Published:** 2016-11-04

**Authors:** Maura N. Laus, Mario Soccio, Michela Alfarano, Antonella Pasqualone, Marcello S. Lenucci, Giuseppe Di Miceli, Donato Pastore

**Affiliations:** a Dipartimento di Scienze Agrarie, degli Alimenti e dell^׳^Ambiente, Università degli Studi di Foggia, Via Napoli, 25, 71122 Foggia, Italy; b Dipartimento di Scienze del Suolo, della Pianta e degli Alimenti, Università di Bari “Aldo Moro”, Via Amendola, 165A, 70126 Bari, Italy; c Dipartimento di Scienze e Tecnologie Biologiche ed Ambientali, Università del Salento, Via Prov.le Lecce-Monteroni, 73100 Lecce, Italy; d Dipartimento di Scienze Agrarie e Forestali, Università degli Studi di Palermo, Viale delle Scienze, 90128 Palermo, Italy; e Fondazione Angelo e Salvatore Lima Mancuso, Piazza Marina, 61, 90100 Palermo, Italy

## Abstract

This article reports experimental data related to the research article entitled “Different effectiveness of two pastas supplemented with either lipophilic or hydrophilic/phenolic antioxidants in affecting serum as evaluated by the novel Antioxidant/Oxidant Balance approach” (M.N. Laus, M. Soccio, M. Alfarano, A. Pasqualone, M.S. Lenucci, G. Di Miceli, D. Pastore, 2016) [1]. Antioxidant status of blood serum of seven healthy subjects was evaluated during four hours after consumption of two functional pastas, supplemented with either bran oleoresin or bran water extract obtained from durum wheat. For comparison, the effect of a non-supplemented reference pasta was also evaluated, as well as the effects of glucose, of the wheat grain dietary supplement Lisosan G, and of the reference pasta consumed together with Lisosan G. Serum antioxidant status was evaluated by measuring both the serum antioxidant capacity, using LOX-FL, ORAC and TEAC methods, and the serum oxidant status, assessed as peroxide level.

**Specifications Table**

**Table:** 

Subject area	*Biochemistry, Food Science*
More specific subject area	*Food Chemistry, Nutrition, Functional Foods*
Type of data	*Table*
How data was acquired	*Fluorimetric and spectrophotometric measurements using a CLARIOstar microplate reader (BMG Labtech, Ortenberg, Germany), a SpectraMax^®^ M5 Multimode Plate Reader (Molecular Devices, Wokingham, UK), and a LS 55 fluorescence spectrometer* (*Perkin Elmer, Waltham, MA, USA)*
Data format	*Analyzed*
Experimental factors	*Venous blood samples were obtained from seven healthy subjects after food intake. After sampling, blood serum was isolated by centrifugation and stored at −80 °C until analysis.*
Experimental features	*Measurements of antioxidant capacity and peroxide level of blood serum of healthy subjects after food intake*
Data source location	*Department of Agricultural, Food and Environmental Sciences, University of Foggia, Foggia, Italy*
Data accessibility	*Data are with this article*

**Value of the data**

• The data can be compared to other data relative to functional foods obtained by other research groups and laboratories using the same assays.
• The data can be used in the development of further studies aimed at evaluating effects of long-term consumption of functional foods.
• The data can be useful as starting point for future investigations aimed at clarifying mechanisms of beneficial effects of foods rich in antioxidants.

## 1 Data

The data presented in this article regard the antioxidant capacity (AC) and peroxide level (PxL) values measured in blood serum of seven healthy subjects at 0, 30, 60, 90, 120 and 240 min after consumption of different foods (Table 1). AC values were obtained by using three different assays: two widely used protocols, such as Trolox Equivalent Antioxidant Capacity (TEAC) [2] and Oxygen Radical Absorbance Capacity (ORAC) [3] methods, and the recently developed Lipoxygenase-Fluorescein (LOX-FL) method [4], derived from the LOX/4-nitroso-*N,N*-dimethylaniline (LOX/RNO) one [5]. PxL values were obtained as described in [1]. Tested foods are differently antioxidant-enriched foods. These included: two functional pastas; a non-supplemented pasta; the wheat antioxidant-rich dietary supplement Lisosan G; glucose; non-supplemented pasta consumed together Lisosan G (see Materials).

## 2 Experimental design, materials and methods

### 2.1 Materials

Foods under study consisted of two supplemented pastas, enriched with either durum wheat (*Triticum durum* Desf.) bran oleoresin (BO) or bran water (BW) extracts showing high content of either lipophilic (tocochromanols, carotenoids) or hydrophilic/phenolic antioxidants, respectively. Other tested foods were: *i*) a non-supplemented reference (R) pasta, consisting of spaghetti produced by Tamma food industry (Foggia, Italy); *ii*) Lisosan G, a nutritional supplement certified by Italian Ministry of Health, produced by Agrisan Company (Larciano, PT, Italy); *iii*) glucose (Baxter, Rome, Italy); *iv*) R pasta consumed together Lisosan G. Production and chemical characterization of BO and BW extracts are described in [6] and [7], respectively. Production and functional, textural and sensory properties of the BO and BW pastas are reported in [8]. For other detailed information about materials, see [1].

### 2.2 Collection of sera from volunteers

As reported in [1], blood samples were obtained from seven healthy subjects (3 women and 4 men aged between 24 and 33 years). Each volunteer attended six testing sessions at 15 days intervals. After 12 h fast, at each session each subject consumed 70 g fresh weight (f.w.) of BW or BO or R pasta, or 20 g f.w. (18 g dry weight, d.w.) of Lisosan G (resuspended in 500 mL of water), or 50 g of glucose (500 mL of 10% solution), or 70 g of R pasta consumed together with 20 g (18 d.w.) of Lisosan G. The subjects consumed one of the test foods within 10–12 min. The subjects assumed pasta together with 500 mL water. Venous blood samples were collected at baseline (*T*_0_) and exactly 30, 60, 90, 120 and 240 min after food consumption. Blood samples were centrifuged at 3000x*g* for 5 min and the resulting serum samples were stored at −80 °C until analysis.

### 2.3 Serum AC determination by means of the LOX-FL, ORAC and TEAC methods

#### 2.3.1 LOX-FL method

The LOX-FL reaction was performed as recently described in [5], modified as in [1]. The quenching of 3׳,6׳-dihydroxy-3H-spiro[2-benzofuran-1,9׳-xanthen]-3-one (fluorescein, FL) coupled to the LOX-1-dependent linoleate peroxidation was continuously monitored (*λ*_ex_=485 nm, *λ*_em_=515 nm) at 37 °C using a LS 55 fluorescence spectrometer (Perkin Elmer, Waltham, MA, USA). The (%) decrease of the rate of LOX-FL reaction measured in the presence of sample in respect of the control was calculated.

#### 2.3.2 ORAC method

The ORAC measurements were performed according to the protocol reported in [3], properly modified as in [1], [5]. The FL fluorescence decay due to peroxyl radicals generated by 2,2’-azobis(2-methylpropionamidine) dihydrochloride (AAPH) thermal decomposition was monitored (*λ*_ex_=483 nm, bandwidth 14 nm; *λ*_*e*m_=530 nm, bandwidth 30 nm) at 37 °C every 30 s by means of a CLARIOstar microplate reader (BMG Labtech, Ortenberg, Germany). To quantify AC, the difference between the area under the fluorescence decay kinetic curve (area under curve, AUC) of sample and the AUC of the blank was calculated.

#### 2.3.3 TEAC method

The TEAC assay described in [2] was applied, modified as in [9], [10]. The diammonium-2,2׳-azino-bis(3-ethylbenzothiazoline-6-sulfonate) radical cation (ABTS^∙+^) was generated by oxidation with potassium persulfate and before use diluted with 5 mM Na-phosphate buffer pH 7.4. The (%) decrease of absorbance at 734 nm (A_734_) measured after 4 min incubation of sample with the ABTS^∙+^ diluted solution was calculated in respect of A_734_ of the inhibited ABTS^∙+^ solution.

For all three AC assays, measurements were carried out in triplicate for at least three different amounts of sample [serum or (±)-6-hydroxy-2,5,7,8-tetramethylchromane-2-carboxylic acid, Trolox, used as standard antioxidant]. A linear dependence of inhibition on serum amount was verified by linear regression analysis of data. AC was calculated by comparing the slope derived by linear regression analysis of the serum with that of the calibration curve obtained by using Trolox as in [11], [12] and expressed as μmol Trolox eq./mL of serum.

### 2.4 Serum PxL determination

Serum PxL was monitored as reported in [1]. The generation of the *N,N*-diethyl-*p*-phenylenediamine (DPPD) radical cation due to alkoxyl radicals generated from hydroperoxides in the presence of Fe^2+^ according to the Fenton reaction was spectrophotometrically monitored at 512 nm and at 37 °C by using a SpectraMax^®^ M5 Multimode Plate Reader (Molecular Devices, Wokingham, UK). Absorbance at 512 nm was evaluated at the end-point (after at least 6 h). Results are expressed in terms of equivalents of H_2_O_2_ using a proper calibration curve. Three different amounts of sera were evaluated in triplicate.

## Figures and Tables

**Table 1:** Antioxidant Capacity (AC), evaluated by LOX-FL, ORAC and TEAC methods, and Peroxide Level (PxL) of serum after consumption of different foods in seven healthy subjects. Serving sizes were: glucose, 50 g; BO (bran oleoresin), BW (bran water extract) and R (reference) pastas, 70 g (fresh weight, f.w.); Lisosan G, 20 g (18 g dry weight, d.w.); R pasta + Lisosan G, 70 g (f.w.) + 20 g (18 d.w.). Data obtained at 0, 30, 60, 90, 120 and 240 min after consumption of each food are reported as mean value±SD (*n*=7 subjects).

	**AC**_**LOX-FL**_**(μmol Trolox eq./mL)**					
	***T***_**0**_	***T***_**30**_	***T***_**60**_	***T***_**90**_	***T***_**120**_	***T***_**240**_

						
Glucose	1.46±0.02	1.30±0.04	1.49±0.03	1.38±0.08	1.26±0.04	1.23±0.03
BO Pasta	1.26±0.07	1.17±0.10	1.46±0.08	1.53±0.04	1.39±0.09	1.16±0.06
BW Pasta	1.31±0.02	1.43±0.08	1.19±0.08	1.41±0.05	1.44±0.01	1.26±0.07
R Pasta	1.51±0.06	1.34±0.01	1.34±0.03	1.18±0.01	1.42±0.01	1.11±0.01
Lisosan G	1.16±0.03	1.60±0.12	1.57±0.12	1.23±0.04	1.52±0.11	1.29±0.12
R Pasta + Lisosan G	1.03±0.07	1.14±0.03	0.89±0.01	1.28±0.02	0.95±0.01	0.97±0.04
	**AC**_**ORAC**_**(μmol Trolox eq./mL)**					
	***T***_**0**_	***T***_**30**_	***T***_**60**_	***T***_**90**_	***T***_**120**_	***T***_**240**_

						
Glucose	12.33±0.41	11.87±0.32	11.31±0.53	10.94±0.54	11.35±0.55	10.49±0.40
BO Pasta	14.57±0.90	13.91±1.15	14.53±1.69	14.49±1.29	14.51±1.10	12.85±1.07
BW Pasta	12.96±0.05	12.74±0.04	13.28±0.05	12.33±0.05	12.41±0.06	11.96±0.06
R Pasta	11.74±0.26	12.56±0.70	12.74±0.29	12.32±0.15	12.07±0.66	12.34±0.33
Lisosan G	16.94±1.60	16.55±1.86	16.85±1.53	16.85±1.31	14.41±1.74	14.87±1.22
R Pasta + Lisosan G	16.25±0.23	12.24±0.75	14.77±0.43	14.84±0.38	12.95±0.23	14.36±0.61
	**AC**_**TEAC**_**(μmol Trolox eq./mL)**					
	***T***_**0**_	***T***_**30**_	***T***_**60**_	***T***_**90**_	***T***_**120**_	***T***_**240**_

						
Glucose	2.55±0.06	2.56±0.05	2.45±0.06	2.50±0.05	2.62±0.08	2.61±0.07
BO Pasta	2.84±0.03	2.84±0.05	2.78±0.08	2.82±0.08	2.92±0.05	2.82±0.12
BW Pasta	2.13±0.02	2.00±0.02	1.98±0.02	1.76±0.06	2.04±0.01	2.02±0.03
R Pasta	2.38±0.07	2.28±0.13	2.28±0.11	2.22±0.13	2.32±0.12	2.29±0.10
Lisosan G	2.40±0.05	2.43±0.03	2.40±0.04	2.31±0.02	2.40±0.04	2.28±0.04
R Pasta + Lisosan G	2.31±0.16	2.32±0.09	2.39±0.18	2.30±0.14	2.42±0.14	2.29±0.17
	**PxL (μmol H**_**2**_**O**_**2**_**eq./mL)**					
	***T***_**0**_	***T***_**30**_	***T***_**60**_	***T***_**90**_	***T***_**120**_	***T***_**240**_

						
Glucose	22.24±1.44	24.59±0.97	25.20±2.06	28.49±2.13	25.20±1.90	26.45±2.12
BO Pasta	21.82±0.90	19.32±0.58	16.24±1.12	19.98±0.86	18.86±0.49	18.16±0.45
BW Pasta	18.51±0.03	22.94±0.08	22.51±0.13	27.91±0.02	23.56±0.09	24.05±0.03
R Pasta	11.77±0.86	13.42±1.34	16.50±1.39	17.99±0.91	16.40±0.24	14.93±1.45
Lisosan G	19.86±1.89	16.61±2.30	21.33±0.90	19.07±1.87	20.42±1.40	21.24±1.34
R Pasta + Lisosan G	21.07±1.87	24.05±1.07	27.37±1.26	20.11±0.52	23.88±1.25	24.22±0.65
